# The Two-Fold Ethical Challenge in the Use of Neural Electrical Modulation

**DOI:** 10.3389/fnins.2019.00678

**Published:** 2019-06-28

**Authors:** Andrea Lavazza

**Affiliations:** Centro Universitario Internazionale, Arezzo, Italy

**Keywords:** neuroethics, aging, enhancement, tDCS—transcranial direct current stimulation, inequalities

The use of electrical stimulation to influence biological functions and/or pathological processes in the body has been recently termed “electroceuticals.” The most commonly used techniques are “neural electroceuticals,” forms of electrical modulation of the brain that seem to represent the new frontier both to treat neurological and psychiatric diseases, when no other effective treatments are available, and to enhance cognitive functions (Kambouris et al., 2014; Reardon, 2014;Miller and Matharu, 2017).

These types of medical interventions have given rise to a wide ethical debate (Pickersgill and Hogle, 2015; Lavazza and Colzato, 2018; Packer et al., 2018). Here I wish to introduce two new challenges bearing important moral implications, which require the careful consideration of the scientific and philosophical community. These challenges can be co-present and can be placed in the same framework of human augmentation and the willingness to go beyond one's own physiological limits. However, it is possible to analytically distinguish them according to their initial conditions and their different scopes, as it will be explained.

The first challenge concerns a possible shift from a mainly therapeutic use of electroceuticals to a use aimed at enhancement. This potential shift is due to the fact that technology has now fulfilled a very ancient human aspiration, that of overcoming one's limits and improving indefinitely. And the effect of this shift could be a segmentation of society between enhanced and non-enhanced individuals, something that goes against the essentially egalitarian project of modern thought (Rawls, 1999; Mason, 2006).

The second challenge concerns the aging tendency and the demographic contraction that characterize European countries and Japan, and which may soon affect other economically developed countries (Lutz et al., 2008; Długosz, 2011; Murray et al., 2018). This trend, over time, will reduce the overall availability of cognitive skills and abilities in those populations, who will have to manage increasingly complex and diversified societies and environments. This mismatch between the needs arising from one's life context and the available resources could push people to resort to electroceuticals as means of strengthening their cognitive abilities, opening up scenarios in which ethical evaluations will have a role to play. Below, I will address these two challenges, giving more space to the first.

## Going Beyond One's Limits

Ever since the *Odyssey*, humans have always desired to alter their minds in a controlled manner through a mix of substances and to go beyond the limits established by brain physiology (Koops et al., 2013). In recent decades, important steps have been taken in this direction, both with new molecules able to act on brain chemistry and with instruments capable of electrically modulating brain activity (Dresler et al., 2018). Scientific consensus on the cognitive enhancement potential of the so-called Non-Invasive Brain Stimulation (NIBS) is not yet unanimous (see Horvath et al., 2015 on one side; Price and Hamilton, 2015 on the other side), but it is undeniable that there is a great investment in research. A growing amount of research studies have produced at least some results in the field, even with different effects at an inter- and intra-individual level. For example, Transcranial Direct Current Stimulation (tDCS) is a form of neurostimulation that so far has been used on healthy subjects to enhance mathematical cognition, reading, memory, mood, learning, perception, decision making, creativity motivation, and moral reasoning (Chi and Snyder, 2012; Callaway, 2013; Meinzer et al., 2013; Snowball et al., 2013; Parkin et al., 2015). The use of NIBS is very often deemed effective by the public due to wide media coverage and Internet ads (Fitz and Reiner, 2015). However, the road to enhancement is now open and more relevant and consistent results may come both from more in-depth knowledge on the functioning of the nervous system and from more performing devices.

What are the consequences of a greater concentration of medical-scientific skills and resources in the field of cognitive neuroenhancement? Medicine is changing, suggests Harari (2016, ch 9), whose line of reasoning is useful here, even though he does not refer to electroceuticals. Somewhat oversimplifying, it can be said that the vocation of medicine, for most of its history, has been to treat the sick, to restore to a better condition those who saw their health deteriorate or were born with a congenital pathology or deficit. Classical Hippocratic medicine has then recently introduced the idea of disease prevention and the notion of combating the symptoms of aging (Bynum, 2008). This was a conceptual and clinical turning point, which has opened the door to the idea of improving the physical and cognitive status of healthy people, thus fulfilling the human aspiration I mentioned earlier, which had not yet been reflected in medical practice.

From an ethical point of view, caring for the sick—at least in principle—is an egalitarian project, because it envisions a level of health which each person can and should ideally reach, despite the limits of medical knowledge and of material resources. This project goes hand in hand with—and derives from—the social and political idea that Christianity and the Enlightenment have brought onto the Western world, according to which all human beings have equal dignity and rights and deserve the same treatment (despite the many exceptions due to material contingencies and the organization of life in society) (Hunt, 2007).

As Harari emphasizes, enhancing those in good health might instead be an elitist project, because it necessarily ignores universal levels of functioning or performance that are applicable to all (More and Vita-More, 2013). Every individual legitimately seeks to gain an advantage over others by exploiting the means made available by medical research to those who can pay for them. Once a certain level of enhancement has been achieved by the whole—or at least by the majority—of the population, the given technology will be available to everyone in terms of both diffusion and cost, and there will be demand for new and further forms of enhancement. These forms of enhancement will be sought by medical-scientific research within the dynamic that always pushes further the frontier of technical knowledge.

Harari's prediction is that the poorest people in the next 50 years will have much better healthcare than today, whereas the health inequality measured in functioning and physical-cognitive performance might get much worse. Strong inequalities have always been present in the history of mankind, even when enhancement was not even contemplated as a possibility. However, for reasons related to technical progress, today there may be no shared interest in ensuring healthcare to the entire population according to the best current standards.

In the twentieth century many states had an interest in, and the possibility of, integrating the masses in the social fabric, also by universally extending the benefits of modern medicine. In fact, there was the need to have millions of soldiers in good health and well-looked after when injured, while the industry benefited from millions of workers in good physical conditions and able to work in factories for many consecutive hours. These were the years when mass hygiene facilities and vaccination campaigns were introduced, and several epidemics were eradicated (cf. Pinker, 2018).

## New Potential Inequalities

The economic and military dynamics of the twenty-first century might be very different from the past. In the era of drones and remote or self-driving military vehicles, mass armies are no longer needed: what is needed are only a few selected super-experts in war technology (Scharre, 2018). The advent of robotics and the use of big data combined with evolving algorithms also make a large part of human work obsolete, so that production tasks can be performed by machines, leaving human beings in charge of more complex activities such as design and supervision (Ford, 2015).

These trends, of which we can already see some indications, could be accentuated and accelerated by the research on cognitive enhancement: the best performing individuals will be the ones to occupy positions of responsibility, as society will want to entrust the most important tasks to those with the best skills (Santoni de Sio et al., 2014). There are also scenarios that seem to come from a dystopian novel and, to the current state of knowledge, are certainly not realistic: such scenarios involve the emergence of superhumans with exceptional physical, emotional and intellectual abilities, which will stand out from the rest of the non-enhanced or less enhanced individuals, because the differences will become not only quantitative but also qualitative, leading to the creation of different groups distinguished by temperament and interests (Bess, 2015).

In fact, quantitative differences concern the increase of cognitive abilities, for example memory. Those who can access these forms of empowerment become high-performing people, who can succeed in the workplace and then improve their condition outperforming those who are not enhanced. Qualitative differences instead are brought on, for example, by genetic modifications thanks to recent techniques such as CRISPR-Cas9 (Lavazza, 2019a). In that case, genetically modified individuals could be different from non-modified individuals in the same way as adults and children or the most educated people and the illiterate ones are different. And social consequences would be predictably very relevant.

The equality project entailed by the material and moral progress of the world so far—which substantially amounts to defeating hunger, diseases and war—aims to guarantee decent living conditions for everyone, so that all people can equally pursue their own life project. Instead, the new goals aiming at overcoming our mortal and uncertain human condition, mainly thanks to technology, can hardly be within everyone's reach and, on the contrary, will often be linked to a privileged condition reserved for a few.

There has certainly been an increase in do-it-yourself use of simple transcranial direct current stimulation (tDCS) devices (Fitz and Reiner, 2015). However, dealing with the use of other latest generation electroceuticals and future more sophisticated devices we will have to address the challenge outlined above. Should we consider prohibiting the use of certain forms of enhancement or should we pursue egalitarian policies, allowing everyone to access electroceuticals? (Lavazza, 2019b). A possible (but debatable) solution is to try to enhance the moral abilities of individuals, to ensure the prevalence of pro-social motives and a general growth of the well-being of individuals and of whole society (Persson and Savulescu, 2012). If this was not possible, one could explore a use of cognitive enhancement according to Rawls's influential view that inequalities are acceptable if they benefit the whole society (Lavazza, 2016). In this sense, cognitively enhancing certain professional figures or public decision-makers will give them a benefit that others will not enjoy but will positively reverberate on the general functioning of society.

## Mandatory Enhancement?

The second challenge concerning electroceuticals is intertwined with the first, while it has a different scope. The processes of scientific and technological innovation on a global scale, along with the phenomena of social complexification, are undergoing continuous acceleration, which will require a greater availability of cognitive skills to manage this complexity and the associated problems (for example, those related to climate change and to the reduction of natural resources). According to Rindermann (2018), however, cognitive abilities in the Western world could go down due to demographic trends. In many nations, fewer births and a longer life expectancy result in a decline in memory, processing speed, attention, creativity and, therefore, in the capacity for innovation. Furthermore, the most educated and cognitively most capable people normally make fewer children.

It is difficult to quantify the phenomenon, both because it is new and because it is still little studied. However, it is plausible to assume that general aging will cause a decrease in the overall cognitive abilities of society. First, there will be more people over the age of 65, while people under the age of 65 will decrease in number. And it is established that “the normal aging process is associated with declines in certain cognitive abilities, such as processing speed and some aspects of memory, language, visuospatial function, and executive functions” (Harada et al., 2013; cf. also Reichman et al., 2010; Salthouse, 2012; Fechner et al., 2019). Secondly, with the number of elderly people increasing, even if the incidence rate remains fixed, the overall percentage of people suffering from diseases that affect cognition will increase. In the United States today there are about 6 million people with dementia; according to some estimates (Alzheimer's Association, 2019) the number will go up to 14 million in 2050, while the overall population will remain stable or grow slightly.

The idea of making enhancement (and cognitive improvement/rehabilitation for aged people) widespread and perhaps even mandatory also comes from arguments that underline how some emergencies cannot be faced with the cognitive and moral endowments that we have today (Lavazza and Reichlin, 2019). Persson and Savulescu (2012), for example, have stated that humans are ethically unfit to face the challenges of the present age. Their argument rests on the fact that today's humankind is facing two kind of threats “generated by the existence of modern scientific technology: the threats of weapons of mass destruction, especially in the hands of terrorist groups, and of climate change and environmental degradation” (Persson and Savulescu, 2012: 1). According to the authors, humans are not morally equipped to address such global problems within a democratic system, especially when it comes to environmental problems. Consequently, cognitive enhancement, understood as the basis of moral betterment, could become the object of policies that make it strongly recommended, encouraged, or mandatory.

In this framework, the classic suggestion is to increase the educational programs that allow for the enhancement of cognitive abilities, which constitute human capital. Specifically, reference is often made to cognitive training programs such as the reasoning training proposed by Klauer and Phye (2008). But if neurocognitive enhancement proves to be safe and effective, it promises to be quicker and more easily administrable to a greater percentage of the population compared to traditional programs, since it does not require the conscious and prolonged effort of the subject. In the case of a real decline in the cognitive abilities of a society as a whole, neurocognitive intervention via neural electrical modulation would become one of the viable options in order to improve the condition of the elderly and compensate for the loss of their cognitive skills and to partially rehabilitate people with degenerative diseases.

This would bring about some ethical questions, as well as the pressure to promote and spread forms of enhancement, and improvement for aged people (since they can only regain the previous performance). In this case, those who want to occupy relevant roles in society might be asked or even forced to undergo the enhancement to make up for the general decline in cognitive abilities. Ethical reflection will then be called to clarify the obligations to be enhanced and the rights of those who do not want to alter the functioning of their mind / brain.

This situation does not exclude the tendency linked to the first challenge that I have illustrated. On the one hand, medicine is concentrating on enhancing a lucky few, who could take advantage of the current dynamics to reverse the pursuit of equality that our societies have been implementing for some time (apart from temporary fluctuations in the distribution of income and wealth). On the other hand, demographic decline and aging may require that more people resort to cognitive enhancement, improvement and rehabilitation to compensate for the decrease in the overall capabilities available to address the complex problems we are facing today.

## Conclusion

These scenarios find their preconditions in trends that are already in place, but which will not be necessarily realized. However, they seem to deserve attention from all those working in the field of electroceuticals and from public decision-makers, that is, all those who can affect future situations. Philosophers and neuroethicists are entrusted with the task of thinking about these scenarios so as not to be unprepared in case they come true.

In the face of these challenges, however, some lines of intervention can already be hypothesized. Faced with the first challenge—that is, the possible shift from a mainly therapeutic use of electroceuticals to a use aimed at enhancement—a stricter regulation of devices must be promoted (Dubljević, 2015; Maslen et al., 2015). Secondly, scientists and clinicians could try to establish guidelines for the use of electroceuticals that should consider not only the safety features but also the possible social consequences of a widespread use of these enhancement techniques. Thirdly, research should be directed primarily at clinical applications, before moving toward the enhancement of healthy subjects.

As for the second challenge, the three recommendations set out above apply as well. More specifically, all operators engaged in medical practices involving electroceuticals should refer to the ethical codes of their respective professions and to international conventions (for example the Oviedo Convention) for the protection of human rights and dignity. All these rules already in force prevent the mandatory administration of medical treatments, except in extraordinary cases that are, or should be, well-specified. It would therefore be important to avoid defining electroceuticals as a non-medical treatment in order to use them only within a legal framework.

Faced with political decisions that could go toward the violation of the rules in force, the scientific community would have the responsibility to highlight the potential risks involved and to actively prevent them as well.

## Author Contributions

The author confirms being the sole contributor of this work and has approved it for publication.

### Conflict of Interest Statement

The author declares that the research was conducted in the absence of any commercial or financial relationships that could be construed as a potential conflict of interest.
